# Supplementary material for: Fms-like tyrosine kinase 3 is a regulator of the cardiac side population in mice
Source: Life Sci Alliance. 2021 Dec 13;5(3):e202101112. doi: 10.26508/lsa.202101112 (PMC8711848; doi:10.26508/lsa.202101112)
Supplement: Supplementary file 1 [file LSA-2021-01112_TableS1.docx]

**Online Supplement**

**Fms-like tyrosine kinase 3 is a regulator of the cardiac side population in mice**

Giacomo Della Verde^1,*^, Michika Mochizuki^1,*^, Vera Lorenz^1^, Julien Roux^1,2^, Lifen Xu^1^, Leandra Ramin-Wright^1^, Otmar Pfister^1,3,#^ and Gabriela M. Kuster^1,3,#^

^1^Department of Biomedicine, University Hospital Basel and University of Basel, Switzerland, ^2^Swiss Institute of Bioinformatics, Basel, Switzerland, and ^3^Department of Cardiology, University Hospital Basel, Basel, Switzerland, ^*^co-first authors; ^#^ co-senior authors

**Supplemental Tables and Figure**

**Supplemental Table 1**

Single gene analysis. Genes that were differentially expressed in flt3L^-/-^ versus wt SP-CPCs. Genes with an adjusted p-value <0.05 are listed.

| **Gene Id** | **Symbol** | **Direction** | **absLog2FC** | **P.Value** | **adj.P.Val** |
| --- | --- | --- | --- | --- | --- |
| ENSMUSG00000038886 | Man2a2 | Down | 0.82 | 3.8E-06 | 4.8E-02 |
| ENSMUSG00000024940 | Ltbp3 | Down | 0.62 | 7.9E-06 | 4.8E-02 |
| ENSMUSG00000031561 | Tenm3 | Down | 0.92 | 1.4E-05 | 4.8E-02 |
| ENSMUSG00000027087 | Itgav | Down | 0.58 | 2.2E-05 | 4.8E-02 |
| ENSMUSG00000036545 | Adamts2 | Down | 1.21 | 2.6E-05 | 4.8E-02 |
| ENSMUSG00000044461 | Shisa2 | Down | 0.71 | 2.7E-05 | 4.8E-02 |
| ENSMUSG00000038894 | Irs2 | Down | 0.98 | 2.7E-05 | 4.8E-02 |
| ENSMUSG00000020902 | Ntn1 | Down | 0.49 | 2.9E-05 | 4.8E-02 |
| ENSMUSG00000038456 | Dennd2a | Down | 0.85 | 3.5E-05 | 4.8E-02 |
| ENSMUSG00000035305 | Ror1 | Down | 0.82 | 3.6E-05 | 4.8E-02 |
| ENSMUSG00000044548 | Dact1 | Down | 0.86 | 3.9E-05 | 4.8E-02 |
| ENSMUSG00000020393 | Kremen1 | Down | 0.38 | 4.7E-05 | 4.9E-02 |
| ENSMUSG00000024059 | Clip4 | Down | 1.05 | 4.8E-05 | 4.9E-02 |
| ENSMUSG00000031548 | Sfrp1 | Down | 1.17 | 5.7E-05 | 5.0E-02 |
| ENSMUSG00000041075 | Fzd7 | Down | 0.53 | 5.8E-05 | 5.0E-02 |
| ENSMUSG00000070436 | Serpinh1 | Up | 1.81 | 6.4E-05 | 5.0E-02 |
| ENSMUSG00000034573 | Ptpn13 | Down | 0.78 | 6.9E-05 | 5.0E-02 |
| ENSMUSG00000021796 | Bmpr1a | Down | 0.82 | 7.0E-05 | 5.0E-02 |
| ENSMUSG00000017466 | Timp2 | Down | 0.58 | 7.3E-05 | 5.0E-02 |
| ENSMUSG00000026676 | Ccdc3 | Down | 1.04 | 7.4E-05 | 5.0E-02 |
