# Supplementary material for: Fms-like tyrosine kinase 3 is a regulator of the cardiac side population in mice
Source: Life Sci Alliance. 2021 Dec 13;5(3):e202101112. doi: 10.26508/lsa.202101112 (PMC8711848; doi:10.26508/lsa.202101112)
Supplement: Supplementary file 2 [file LSA-2021-01112_TableS2.docx]

**Online Supplement**

**Fms-like tyrosine kinase 3 is a regulator of the cardiac side population in mice**

Giacomo Della Verde^1,*^, Michika Mochizuki^1,*^, Vera Lorenz^1^, Julien Roux^1,2^, Lifen Xu^1^, Leandra Ramin-Wright^1^, Otmar Pfister^1,3,#^ and Gabriela M. Kuster^1,3,#^

^1^Department of Biomedicine, University Hospital Basel and University of Basel, Switzerland, ^2^Swiss Institute of Bioinformatics, Basel, Switzerland, and ^3^Department of Cardiology, University Hospital Basel, Basel, Switzerland, ^*^co-first authors; ^#^ co-senior authors

**Supplemental Tables and Figure**

**Supplemental Table 2**

Gene set enrichment analysis. Hallmark signature gene sets that are differentially expressed in flt3L^-/-^ versus wt SP-CPCs. Gene sets with an adjusted p-value <0.05 are listed.

| **Gene Set** | **NGenes** | **Direction** | **absLog2FC** | **P.Value** | **adj.P.Val** |
| --- | --- | --- | --- | --- | --- |
| HALLMARK EPITHELIAL MESENCHYMAL TRANSITION | 180 | Down | 0.45 | 5.8E-11 | 2.9E-09 |
| HALLMARK INTERFERON ALPHA RESPONSE | 101 | Up | 0.25 | 1.7E-08 | 4.2E-07 |
| HALLMARK MYC TARGETS V1 | 194 | Up | 0.15 | 1.6E-05 | 2.6E-04 |
| HALLMARK DNA REPAIR | 151 | Up | 0.17 | 5.6E-04 | 7.0E-03 |
| HALLMARK OXIDATIVE PHOSPHORYLATION | 200 | Up | 0.12 | 1.0E-03 | 1.0E-02 |
| HALLMARK INTERFERON GAMMA RESPONSE | 198 | Up | 0.30 | 1.2E-03 | 1.0E-02 |
| HALLMARK MTORC1 SIGNALING | 195 | Up | 0.22 | 3.2E-03 | 2.1E-02 |
| HALLMARK UV RESPONSE DN | 139 | Down | 0.30 | 3.4E-03 | 2.1E-02 |
| HALLMARK UNFOLDED PROTEIN RESPONSE | 111 | Up | 0.16 | 8.6E-03 | 4.8E-02 |
