# Supplementary material for: Fms-like tyrosine kinase 3 is a regulator of the cardiac side population in mice
Source: Life Sci Alliance. 2021 Dec 13;5(3):e202101112. doi: 10.26508/lsa.202101112 (PMC8711848; doi:10.26508/lsa.202101112)
Supplement: Supplementary file 3 [file LSA-2021-01112_TableS3.docx]

**Online Supplement**

**Fms-like tyrosine kinase 3 is a regulator of the cardiac side population in mice**

Giacomo Della Verde^1,*^, Michika Mochizuki^1,*^, Vera Lorenz^1^, Julien Roux^1,2^, Lifen Xu^1^, Leandra Ramin-Wright^1^, Otmar Pfister^1,3,#^ and Gabriela M. Kuster^1,3,#^

^1^Department of Biomedicine, University Hospital Basel and University of Basel, Switzerland, ^2^Swiss Institute of Bioinformatics, Basel, Switzerland, and ^3^Department of Cardiology, University Hospital Basel, Basel, Switzerland, ^*^co-first authors; ^#^ co-senior authors

**Supplemental Table 3**

Gene set enrichment analysis. Gene ontology gene sets that are differentially expressed in flt3L^-/-^ versus wt SP-CPCs. The top 20 gene sets according to adjusted p-value are listed.

| **Gene Set** | **NGenes** | **Direction** | **absLog2FC** | **P.Value** | **adj.P.Val** |
| --- | --- | --- | --- | --- | --- |
| GO_EXTRACELLULAR MATRIX STRUCTURAL CONSTITUENT | 115 | Down | 0.52 | 4.7E-18 | 4.4E-14 |
| GO_EXTRACELLULAR MATRIX STRUCTURAL CONSTITUENT CONFERRING TENSILE STRENGTH | 31 | Down | 0.71 | 2.2E-16 | 1.0E-12 |
| GO_COLLAGEN TRIMER | 55 | Down | 0.55 | 3.6E-14 | 1.1E-10 |
| GO_EXTRACELLULAR MATRIX | 386 | Down | 0.47 | 1.4E-12 | 3.0E-09 |
| GO_COMPLEX OF COLLAGEN TRIMERS | 17 | Down | 0.73 | 1.6E-12 | 3.0E-09 |
| GO_COLLAGEN CONTAINING EXTRACELLULAR MATRIX | 308 | Down | 0.47 | 4.7E-11 | 7.3E-08 |
| GO_EXTRACELLULAR STRUCTURE ORGANIZATION | 311 | Down | 0.45 | 7.7E-11 | 1.0E-07 |
| GO_FIBRILLAR COLLAGEN TRIMER | 10 | Down | 0.79 | 6.0E-10 | 7.1E-07 |
| GO_WNT PROTEIN BINDING | 26 | Down | 0.52 | 1.2E-09 | 1.3E-06 |
| GO_REGULATION OF NATURAL KILLER CELL CHEMOTAXIS | 10 | Down | 0.97 | 3.5E-09 | 3.2E-06 |
| GO_GLYCOSAMINOGLYCAN BINDING | 146 | Down | 0.42 | 3.7E-09 | 3.2E-06 |
| GO_NATURAL KILLER CELL CHEMOTAXIS | 12 | Down | 0.89 | 4.4E-09 | 3.5E-06 |
| GO_COLLAGEN FIBRIL ORGANIZATION | 47 | Down | 0.56 | 3.2E-08 | 1.8E-05 |
| GO_HEPARIN BINDING | 107 | Down | 0.43 | 6.0E-08 | 3.1E-05 |
| GO_PROTEIN COMPLEX INVOLVED IN CELL ADHESION | 35 | Down | 0.57 | 8.7E-08 | 3.9E-05 |
| GO_REGULATION OF LYMPHOCYTE CHEMOTAXIS | 24 | Down | 0.66 | 8.7E-08 | 3.9E-05 |
| GO_REGULATION OF CELLULAR AMINO ACID METABOLIC PROCESS | 57 | Up | 0.17 | 1.5E-07 | 5.7E-05 |
| GO_HOMOPHILIC CELL ADHESION VIA PLASMA MEMBRANE ADHESION MOLECULES | 116 | Down | 0.37 | 1.9E-07 | 7.0E-05 |
| GO_ATP SYNTHESIS COUPLED ELECTRON TRANSPORT | 87 | Up | 0.17 | 2.0E-07 | 7.0E-05 |
| GO_ANTIGEN PROCESSING AND PRESENTATION OF EXOGENOUS PEPTIDE ANTIGEN VIA MHC CLASS I | 69 | Up | 0.22 | 2.0E-07 | 7.0E-05 |
