# Supplementary material for: Fms-like tyrosine kinase 3 is a regulator of the cardiac side population in mice
Source: Life Sci Alliance. 2021 Dec 13;5(3):e202101112. doi: 10.26508/lsa.202101112 (PMC8711848; doi:10.26508/lsa.202101112)
Supplement: Supplementary file 4 [file LSA-2021-01112_TableS4.docx]

**Online Supplement**

**Fms-like tyrosine kinase 3 is a regulator of the cardiac side population in mice**

Giacomo Della Verde^1,*^, Michika Mochizuki^1,*^, Vera Lorenz^1^, Julien Roux^1,2^, Lifen Xu^1^, Leandra Ramin-Wright^1^, Otmar Pfister^1,3,#^ and Gabriela M. Kuster^1,3,#^

^1^Department of Biomedicine, University Hospital Basel and University of Basel, Switzerland, ^2^Swiss Institute of Bioinformatics, Basel, Switzerland, and ^3^Department of Cardiology, University Hospital Basel, Basel, Switzerland, ^*^co-first authors; ^#^ co-senior authors

**Supplemental Tables and Figure**

**Supplemental Table 4**

Gene set enrichment analysis. Gene ontology gene sets that are differentially expressed in flt3L^-/-^ versus wt SP-CPCs, selected according to function within the cardiovascular system and with an adjusted p-value <0.05 are listed.

| **Gene Set^1^** | **NGenes** | **Direction** | **absLog2FC** | **PValue** | **adj.P.Val** |
| --- | --- | --- | --- | --- | --- |
| GO_REGULATION OF HEART MORPHOGENESIS | 32 | Down | 0.36 | 1.0E-04 | 4.2E-03 |
| GO_HEART FIELD SPECIFICATION | 11 | Down | 0.40 | 3.0E-04 | 8.8E-03 |
| GO_HEART FORMATION | 22 | Down | 0.37 | 3.6E-04 | 9.6E-03 |
| GO_POSITIVE REGULATION OF HEART GROWTH | 32 | Down | 0.37 | 3.7E-04 | 9.9E-03 |
| GO_HEART VALVE DEVELOPMENT | 61 | Down | 0.32 | 6.8E-04 | 1.4E-02 |
| GO_REGULATION OF HEART GROWTH | 56 | Down | 0.33 | 7.8E-04 | 1.5E-02 |
| GO_HEART GROWTH | 76 | Down | 0.31 | 8.4E-04 | 1.6E-02 |
| GO_CELL MIGRATION INVOLVED IN HEART DEVELOPMENT | 12 | Down | 0.36 | 4.4E-03 | 4.6E-02 |

| **Gene Set^2^** | **NGenes** | **Direction** | **absLog2FC** | **PValue** | **adj.P.Val** |
| --- | --- | --- | --- | --- | --- |
| GO_CENTRAL NERVOUS SYSTEM PROJECTION NEURON AXONOGENESIS | 22 | Down | 0.47 | 5.8E-06 | 6.3E-04 |
| GO_REGULATION OF HEMATOPOIETIC STEM CELL DIFFERENTIATION | 69 | Up | 0.20 | 3.3E-05 | 2.0E-03 |
| GO_SKELETAL SYSTEM DEVELOPMENT | 375 | Down | 0.32 | 3.5E-05 | 2.0E-03 |
| GO_POSITIVE REGULATION OF STEM CELL DIFFERENTIATION | 16 | Down | 0.45 | 7.1E-05 | 3.2E-03 |

| **Gene Set^3^** | **NGenes** | **Direction** | **absLog2FC** | **PValue** | **adj.P.Val** |
| --- | --- | --- | --- | --- | --- |
| GO_POSITIVE REGULATION OF VASCULAR ASSOCIATED SMOOTH MUSCLE CELL MIGRATION | 14 | Down | 0.37 | 5.6E-04 | 1.3E-02 |
| GO_VASCULAR ASSOCIATED SMOOTH MUSCLE CONTRACTION | 20 | Down | 0.41 | 8.1E-04 | 1.6E-02 |

| **Gene Set^4^** | **NGenes** | **Direction** | **absLog2FC** | **PValue** | **adj.P.Val** |
| --- | --- | --- | --- | --- | --- |
| GO_CORONARY VASCULATURE DEVELOPMENT | 42 | Down | 0.30 | 3.5E-03 | 4.0E-02 |

^1^search term “heart”; ^2^search term “stem”; ^3^search term “vascular”; ^4^search term “coronary”.
