# Supplementary material for: Fms-like tyrosine kinase 3 is a regulator of the cardiac side population in mice
Source: Life Sci Alliance. 2021 Dec 13;5(3):e202101112. doi: 10.26508/lsa.202101112 (PMC8711848; doi:10.26508/lsa.202101112)
Supplement: Supplementary file 5 [file LSA-2021-01112_TableS5.docx]

**Online Supplement**

**Fms-like tyrosine kinase 3 is a regulator of the cardiac side population in mice**

Giacomo Della Verde^1,*^, Michika Mochizuki^1,*^, Vera Lorenz^1^, Julien Roux^1,2^, Lifen Xu^1^, Leandra Ramin-Wright^1^, Otmar Pfister^1,3,#^ and Gabriela M. Kuster^1,3,#^

^1^Department of Biomedicine, University Hospital Basel and University of Basel, Switzerland, ^2^Swiss Institute of Bioinformatics, Basel, Switzerland, and ^3^Department of Cardiology, University Hospital Basel, Basel, Switzerland, ^*^co-first authors; ^#^ co-senior authors

**Supplemental Tables and Figure**

**Supplemental Table 5**

List of antibodies and markers used for flow cytometry, western blotting, and immunocyto- and -histochemistry.

| **Product Name** | **Company** | **Cat. No.** | **Amount** | **Assay** |
| --- | --- | --- | --- | --- |
| FITC rat anti-mouse Ly-6A/E (Sca-1) | BD Biosciences | #557405 | 0.6 µg/10^7^ cells | FC* |
| FITC rat IgG2a k isotype control | BD Biosciences | #554688 | 0.6 µg/10^7^ cells | FC |
| APC rat anti-mouse CD31 | BD Biosciences | #551262 | 0.25 µg/10^7^ cells | FC |
| Alexa fluor 647 rat anti-mouse CD34 | BD Biosciences | #560230 | 0.25 µg/10^7^ cells | FC |
| APC rat IgG2a k isotype control | BD Biosciences | #553932 | 0.25 µg/10^7^ cells | FC |
| Pacific blue rat anti-mouse CD45 | Biolegend | #103125 | 0.6 µg/10^7^ cells | FC |
| Pacific blue rat IgG2b k isotype control | Biolegend | #400627 | 0.6 µg/10^7^ cells | FC |
| 7-Aminoactinomycin D (7-ADD) | ThermoFisher | #A1310 | 0.15 µg/10^6^ cells | FC |
| Rabbit anti-Akt | Cell Signaling | #9272 | 1/7000 | WB |
| Rabbit anti-phospho-Akt (Ser473) | Cell Signaling | #4058 | 1/2000 | WB |
| Rabbit anti-Calponin | Cell Signaling | #17819 | 1/1000 | WB |
| Mouse anti-GAPDH | Merck | #G8795 | 1/10000 | WB |
| Rabbit anti-human von Willebrand Factor (vWF) | Abcam | #ab6994 | 1/100 | ICC |
| Goat anti-rabbit IgG, Alexa Fluor 546 | ThermoFisher | #A11010 | 1/800 | ICC |
| DAPI | Merck | #D1306 | 1/1000 | ICC |
| DyLight 594 labelled IB4 | Vector labs | #DL-1207 | 1/25 | IHC |
| FITC-conjugated Lectin (Wheat Germ Agglutinin, WGA) | Merck | #L4895 | 1/100 | IHC |

*FC: Flowcytometry; WB: Western Blot; ICC: Immunocytochemistry; IHC: Immunohistochemistry
