# Supplementary material for: Fms-like tyrosine kinase 3 is a regulator of the cardiac side population in mice
Source: Life Sci Alliance. 2021 Dec 13;5(3):e202101112. doi: 10.26508/lsa.202101112 (PMC8711848; doi:10.26508/lsa.202101112)
Supplement: Supplementary file 6 [file LSA-2021-01112_TableS6.docx]

**Online Supplement**

**Fms-like tyrosine kinase 3 is a regulator of the cardiac side population in mice**

Giacomo Della Verde^1,*^, Michika Mochizuki^1,*^, Vera Lorenz^1^, Julien Roux^1,2^, Lifen Xu^1^, Leandra Ramin-Wright^1^, Otmar Pfister^1,3,#^ and Gabriela M. Kuster^1,3,#^

^1^Department of Biomedicine, University Hospital Basel and University of Basel, Switzerland, ^2^Swiss Institute of Bioinformatics, Basel, Switzerland, and ^3^Department of Cardiology, University Hospital Basel, Basel, Switzerland, ^*^co-first authors; ^#^ co-senior authors

**Supplemental Tables and Figure**

**Supplemental Table 6**

List of compounds and compositions of culture media.

| **Product Name** | **Company** | **Cat. No.** | **Amount** |
| --- | --- | --- | --- |
|  |  |  |  |
| **Expansion Medium 1 (EM1)** |  |  |  |
| MEM α GlutaMAX Supplement | ThermoFisher | #32561 | 500 mL |
| Hepes | ThermoFisher | #15630 | 25 mM |
| Penicillin-Streptomycin (P/S) | ThermoFisher | #15140 | 1% |
| Fetal Bovine Serum (FBS) | Hyclone | #SH30071 | 20% |
| Sodium Pyruvate | ThermoFisher |  | 1% |
|  |  |  |  |
| **Expansion Medium 2 (EM2)** |  |  |  |
| Iscove's Modified Dulbecco's Medium (IMDM) | ThermoFisher | #12440 | 35% |
| Dulbecco's Modified Eagle's Medium (DMEM)/Nutrient Mixture F12 Ham | Millipore-Sigma | #D8437 | 65% |
| Penicillin-Streptomycin (P/S) | ThermoFisher | #15140122 | 1% |
| Fetal Bovine Serum (FBS) | Hyclone | #SH30071 | 3.5%* |
| L-Glutamine | ThermoFisher | #25030 | 2 mM |
| L-Glutathione reduced | Merck | #G6013 | 0.2 nM |
| B27 Supplement | ThermoFisher | #17504044 | 1.3% |
| Recombinant Murine Cardiotrophin-1 | Peprotech | #250-25 | 0.65 ng/mL |
| Thrombin | Diagontech AG, CH | #100-125 | 0.0005 U/mL |
| Recombinant Human Epidermal Growth Factor (EGF) | Peprotech | #AF-100-15 | 6.5 ng/mL |
| Recombinant Basic Fibroblast Growth Factor (FGF) | Peprotech | #AF-100-18B | 13 ng/mL |
|  |  |  |  |
| **Smooth muscle differentiation medium (SMD)** |  |  |  |
| Iscove's Modified Dulbecco's Medium (IMDM) | ThermoFisher | #12440 | 35% |
| Dulbecco's Modified Eagle's Medium (DMEM)/Nutrient Mixture F12 Ham | Merck | #D8437 | 65% |
| Penicillin-Streptomycin (P/S) | ThermoFisher | #15140122 | 1% |
| Fetal Bovine Serum (FBS) | Hyclone | #SH30071 | 3.5%* |
| L-Glutamine | ThermoFisher | #25030 | 2 mM |
| L-Glutathione reduced | Merck | #G6013 | 0.2 nM |
| B27 Supplement | ThermoFisher | #17504044 | 1.3% |
| Recombinant Murine Cardiotrophin-1 | Peprotech | #250-25 | 0.65 ng/mL |
| Thrombin | Diagontech AG, CH | #100-125 | 0.0005U/mL |
| Recombinant Human Platelet-Derived Growth Factor BB (PDGF-BB) | Peprotech | #100-14B | 10ng/mL |
|  |  |  |  |
| **Endothelial differentiation medium (EGM2)** |  |  |  |
| Fibronectin | Merck | #F4759 | 10 µg/mL |
| EGM-2 Endothelial Cell Growth Medium-2 BulletKit, containing: | Lonza | #CC-3162 |  |
| 1. EBM-2 Endothelial Cell Growth Basal Medium-2 | Lonza | #CC-3156 | 500mL |
| 1. EGM-2 Endothelial SingleQuots kit, containing: | Lonza | #CC-4176 |  |
| Fetal Bovine Serum (FBS) |  |  | 2% |
| Human epidermal growth factor (hEGF) |  |  | 0.5mL |
| Vascular endothelial growth factor (VEGF) |  |  | 0.5mL |
| R3-Insulin like growth factor (R3-IGF-1) |  |  | 0.5mL |
| Ascorbic acid |  |  | 0.5mL |
| Hydrocortisone |  |  | 0.2mL |
| Human Fibroblast growth factor-basic (hFGF-β) |  |  | 2mL |
| Heparin |  |  | 0.5mL |
| Gentamicin/Amphotericin-B |  |  | 0.5mL |

*lowered to 0.1% during 24 hours for lineage induction.
