# Supplementary material for: Fms-like tyrosine kinase 3 is a regulator of the cardiac side population in mice
Source: Life Sci Alliance. 2021 Dec 13;5(3):e202101112. doi: 10.26508/lsa.202101112 (PMC8711848; doi:10.26508/lsa.202101112)
Supplement: Supplementary file 7 [file LSA-2021-01112_TableS7.docx]

**Online Supplement**

**Fms-like tyrosine kinase 3 is a regulator of the cardiac side population in mice**

Giacomo Della Verde^1,*^, Michika Mochizuki^1,*^, Vera Lorenz^1^, Julien Roux^1,2^, Lifen Xu^1^, Leandra Ramin-Wright^1^, Otmar Pfister^1,3,#^ and Gabriela M. Kuster^1,3,#^

^1^Department of Biomedicine, University Hospital Basel and University of Basel, Switzerland, ^2^Swiss Institute of Bioinformatics, Basel, Switzerland, and ^3^Department of Cardiology, University Hospital Basel, Basel, Switzerland, ^*^co-first authors; ^#^ co-senior authors

**Supplemental Tables and Figure**

**Supplemental Table 7**

List of primers.

| **Gene** | **Forward 5’-3** | **Reverse 5’-3** |
| --- | --- | --- |
| Von Willebrand Factor (vWF) | GATGGAGGGGAGCTTGAACTG | CGACTCCACCACCTCAAAGTG |
| Tie2 | TGCCCTCCTGGGTTTATGG | GGTCCTGCCAAATGTGTGC |
